# Distribution of Drug Substances in Solid Lipid Microparticles (SLM)—Methods of Analysis and Interpretation

**DOI:** 10.3390/pharmaceutics14020335

**Published:** 2022-01-31

**Authors:** Eliza Wolska, Marta Brach

**Affiliations:** 1 Department of Pharmaceutical Technology, Medical University of Gdansk, Hallera 107, 80-416 Gdansk, Poland; 2 Student Chapter of the International Society of Pharmaceutical Engineering (ISPE), Hallera 107, 80-416 Gdansk, Poland; martabrach@gumed.edu.pl

**Keywords:** solid lipid microparticles, microspheres, drug distribution, entrapment efficiency, drug loading, aqueous phase, interphase, lipid core, solid lipid nanoparticles, cyclosporine

## Abstract

The incorporation of drug substances into the matrix of solid lipid microparticles (SLM) is critical to providing effects such as prolonged release, taste masking, and protection of the labile API. Currently, a commonly used method of characterizing multi-compartment lipid systems, such as SLM, is to determine entrapment efficiency (EE) and drug loading (DL) parameters, but this is not sufficient for understanding the localization of API either in the core or on the surface of the microspheres. The main objective of the research was to study the distribution of API in an aqueous dispersion of SLM in order to distinguish between the API incorporated in the lipid matrix and localized in the superficial region (interphase) and to refer the obtained results to the EE and DL parameters. SLM dispersions (10–30% of the lipid) with four model drug substances, i.e., cyclosporine, clotrimazole, diclofenac sodium and hydrocortisone, were prepared and investigated. In the first stage, the experiments were designed to optimize the method of extracting the API fraction localized on the SLM surface by shaking the dispersions with methanol. The fraction dissolved in the aqueous phase was obtained by ultrafiltration of SLM dispersions. Total drug content and the concentration in the separated phases were determined by the HPLC method. The obtained results were compared with the EE and DL parameters. Selected SLM dispersions were tested both before and after thermal sterilization. Short-term shaking of SLM dispersion with methanol does not damage the lipid matrix and allows the API fraction localized on the SLM surface to be extracted, the result of which was the determination of API distribution between lipid matrix, interphase and aqueous phase. It was found that the majority of API represented by EE value was localized on the surface of SLM. Only for cyclosporine was the incorporation of drug molecules in the lipid core very effective (up to 48%), while for other drug substances only 1–21% was found in the lipid core of SLM. A clear influence of the sterilization process on the distribution of API within the microparticles was found. The presented studies showed that the characterization of multi-compartment SLM dispersions solely on the basis of EE and DL values, is insufficient. The proposed new distribution test method enables the localization of API to be demonstrated within the microspheres, with the quantitative characteristics of the drug fraction incorporated in the lipid matrix and the fraction associated with the surface of the lipid matrix. The proposed new method allows the influence of the sterilization process on the changes in the API distribution within the lipospheres to be evaluated. Such characteristics provide new opportunities for the development and use of this dosage form as a carrier providing prolonged release and other aforementioned advantages.

## 1. Introduction

Solid lipid microparticles (SLM) are drug carriers measured in micrometers, made of lipids with a melting point above human body temperature. They can be administered in the form of an aqueous dispersion or, after drying, may be presented as a fine powder [1,2,3,4,5,6,7,8]. In terms of composition and some properties, they are similar to SLN (solid lipid nanoparticles), formulations that are much more frequently tested and described [9,10,11].

Lipids are physiologically compatible and biodegradable constituents, which makes SLM a dosage form that is well tolerated. SLM dispersions can be produced without organic solvents, in a simple process, even on industrial scale, and at relatively low costs [4,12]. In addition, they can be sterilized by autoclaving [2,13].

The use of lipid microspheres as drug carriers is associated with the solid nature of the lipid matrix, which enables prolonged drug release, protection from degradation (e.g., hydrolysis), masking drug taste and even improving bioavailability [4,12,14].

SLM are produced by emulsification method, followed by cooling the resulting O/W emulsion. The emulsion and final SLM dispersion is stabilized by a surfactant that is located at the interphase as it is associated with the surface of SLM [14]. The active pharmaceutical ingredient (API) may be located in different regions of the SLM dispersion: incorporated in the lipid core of SLM, on their surface or in the aqueous phase of the dispersion. In the latter, API is dissolved but undesirable precipitation may also occur. The efficacy of drug incorporation in the lipid matrix determines not only the dose but also the use of SLM as a drug carrier providing prolonged release. As is known, the drug distribution in delivery carrier was found to be correlated with the drug release profiles and it affects all other properties of the formulation [4,9,10,14,15].

The amount of the drug incorporated in the SLM is commonly characterized by two parameters: EE (entrapment efficiency or encapsulation efficiency) and DL (drug loading capacity). Encapsulation efficiency is the amount of the drug incorporated (successfully entrapped) in the particles divided by its overall amount added to the formulation. Loading capacity is the amount of drug loaded per unit weight of the total carrier system or lipid in SLM. To calculate these parameters in SLM dispersion the aqueous phase is separated (by dialysis, ultracentrifugation or membrane filtration), concentration of the drug dissolved is determined and this fraction is considered as a free drug, while the rest is incorporated in the microparticles [3,4,5,12,16]. When SLM is in a dry form, a direct measurement of the drug content in the particles can be determined after extraction following the heating step above the melting point of the lipid used [6,8,12,17].

The EE and DL parameters are usually considered as measures of the application potential of the drug carrier system. However, the drug can be localized in different regions of the particles, which the EE and DL results no longer distinguish [18,19]. Some attempts were also made to determine the fraction present on the outer layer of microparticles by using an ultrafiltration centrifugation method after suspending SLM in water [20]. However, the use of water to dissolve practically insoluble in water diflunisal and poorly soluble salicylic acid seems to be a method of limited utility.

Further and more precise information on drug effective encapsulation in the cores of SLM or its surface localization can be obtained by employing imaging techniques, such as XPS, SEM, confocal laser scanning microscopy, Raman spectroscopy and fluorescence spectroscopy, fluorescence anisotropy measurements and others [12,15,21,22]. However, these techniques are relatively more demanding, complicated and not always available. Moreover, they do not provide information on the quantitative distribution of API between the lipid matrix and the surface of the particles.

The presence of a significant fraction of API on the surface of the microparticles or nanoparticles was already reported in numerous publications [4,18,19,22,23,24]. These reports were most often based on the obtained release profiles (burst release effect), as well as the above-mentioned imaging techniques. However, so far no quantitative study has been presented, which would allow a detailed determination of the drug distribution, considering the structural domains in lipospheres (between fraction localized on the surface and in the lipid core). In dispersed systems, the surface-located API is anchored in the interphase formed by the molecules of the surfactant stabilizing the system. This fraction is responsible for the burst release, and does not allow taste to be masked effectively or chemical degradation to be eliminated if these are the goals of the system. Thus, EE and DL parameters, not taking into consideration the phenomena on the particles surface, are insufficient for determining how effective drug incorporation is in SLM.

For the purpose of the presented research, it is important to specify the nomenclature used, referring to the surface layers of lipid microspheres. The nomenclature distinguishes the following terms: interphase and interface, which are used when describing lipospheres dispersion, emulsions and other particulate systems, often as a substitute and without explanation [25,26]. Therefore, in this paper the following definitions of both terms have been adopted: “interface” as the two dimensional boundary between two surfaces (lipid–water) and “interphase” as the three dimensional region between two phases (lipid–cloud of surfactant–water).

In this study, we attempted to assess the distribution of model drug substances to individual phases of the aqueous SLM dispersion, with particular emphasis on the distribution of API within the lipid microspheres. For this purpose, a technique of shaking the dispersion of SLM with an organic solvent (methanol) was proposed and developed, allowing extraction of API from the interphase without dissolving and damaging the lipid matrix of the microspheres. This procedure is simple, allows quantification of the API and has not been used so far. Before using the proposed method, the effect of methanol on the matrix of lipid microspheres was assessed, using not only microscopic imaging, but also particle size distribution. The optimized technique was used to test SLM dispersion with four selected drug substances in different concentrations. The three fractions of API, i.e., incorporated in the lipid matrix, bound to the microspheres but located on the surface (interphase), and dissolved in the aqueous phase of the dispersion, were quantified and the results were compared with the EE and DL values obtained with a standard method.

## 2. Materials and Methods

### 2.1. Materials

Cyclosporine A (CsA) was purchased from LC Laboratories (Boston, MA, USA), diclofenac sodium (DNa) and clotrimazole (KL) from Amoli Organics (Mumbai, India) and hydrocortisone (HC) from PPH Galfarm (Cracow, Poland). Compritol 888 ATO (glyceryl behenate) and Precirol ATO 5 (glyceryl palmitostearate) were obtained from Gattefossé (Saint-Priest, France), while Miglyol 812 (medium chain triglycerides) was obtained from Caelo Caesar and Loretz (Hilden, Germany). Stearic acid and Tween 80 (polysorbate 80) were purchased from Sigma-Aldrich (St. Louis, MO, USA) while methanol and acetonitrile from Merck (Darmstadt, Germany). All other chemicals used were of analytical reagent grade.

### 2.2. Solubility of Drug Substances in Lipids

The solubility of tested drug substances in solid lipids—Compritol (C), stearic acid (SA) and in the blend (PM) of Precirol (P) with liquid lipid, Miglyol (M) in the ratio 4:1—was studied semi-quantitatively. Small amounts of pure active substance were added in portions (until incomplete dissolution of the next added portion of 5 mg was observed) to melted lipids at 80 ± 1 °C and stirred with a magnetic stirrer. The tests were repeated (in order to confirm the amount of the substance that could be dissolved in the lipid) by adding the amount of the active substance determined in the previous test in one portion to the molten lipid and observing its complete dissolution. Solubility was estimated visually in the melted lipids (in a test tube) and after cooling (microscopic observations in thin layers).

### 2.3. Preparation of SLM Dispersions

The composition of all tested API-loaded SLM formulations is listed in Table 1. The majority of prepared dispersions contained 10% (*w*/*w*) of the lipid phase, but some contained 20% or 30% of the lipids. The matrix-forming lipid was usually Compritol, stearic acid or a mixture of solid Precirol with liquid Miglyol. The tested SLM dispersions contained four different active substances in a wide range of concentrations: from 0.1% to 2% (*w*/*w*), which in relation to the lipid content was from 1% to 20%. Tween 80 was used as an emulsifying agent and the isotonicity was adjusted with glycerol.

SLM formulations were prepared using a hot emulsification method, followed by cold re-solidification [13,18]. The mixing process of the lipid phase (lipid, API) with the aqueous phase (water, surfactant and glycerol) was performed at 80 °C using a high-shear mixer Ultra-Turrax (T25 Janke-Kunkel, IKA Labortechnik, Germany) at the speed of 8000 rpm for 5 min. After cooling in an ice bath (30 min), the dispersions were stored in a refrigerator. Selected SLM dispersions were also sterilized at 121 °C for 15 min in an autoclave. After thermal sterilization, hot vials with SLM were mixed for 1 min using vortex.

### 2.4. Characterization of SLM

The methods for assessment of SLM properties were described in detail previously [13,18,19,27]. The microspheres were observed using the optical microscope and scanning electron microscope (see Section 2.5). The particle size distribution in SLM dispersions was determined by laser diffraction method (Beckman-Coulter LS 13 320, Indianapolis, IN, USA). Universal Liquid Module (ULM) was used for the measurements, in which the SLM dispersion was diluted in water, in the sample cell, before the measurement. The obtained results were recorded in the form of a graphs and parameters such as *d_10_*, *d_50_*, *d_90_*, determined as measures of maximum diameter of 10%, 50% and 90% of the detected particles, respectively. The pH of tested formulations was measured directly in the SLM dispersion using a pH meter (Orion, Boston, MA, USA) and osmotic pressure was determined on the 0.15 mL aliquot of the sample by the freezing-point method using the Microosmometer Digital (Knauer, Berlin, Germanny). Zeta potential was determined from the electrophoretic mobility measured with Zetasizer Nano ZS (Malvern Instruments, Worcestershire, UK), after dilution (1:1000) in water at 25 °C.

### 2.5. Optical Microscopy and Scanning Electron Microscopy

Freshly prepared SLM dispersions were observed under optical microscope (Nikon, Eclipse 50i, Nikon Corporation, Tokyo, Japan).

The effect of methanol on SLM morphology was assessed after preparing the samples in the same way as described in Section 2.7. SLM dispersions were diluted 1:5 (*w*/*v*) with methanol, vortexed and centrifuged. Methanol was removed by decantation and the sediment of microspheres was dispersed in water or a 3% polysorbate 80 solution and vortexed for 1 min. The particle size distribution was made with optical microscope software (NIS-Elements BR 30 Nikon, Tokyo, Japan).

The degree of SLM dissolution in methylene chloride was also assessed by microscopic observations of placebo SLM dispersed in methylene chloride at a ratio of 1:1, 6:1 and 25:1 (*w*/*v*). The micrographs of bulk lipid powder after shaking with tested solvents (methanol, methylene chloride, 1:5 *w*/*v*) were also recorded.

To visualize morphology and SLM surface, a scanning electron microscope Phenom Pro (Phenom World Thermo Fisher, Eindhoven, Netherlands) was employed. Standard sample holder and a carbon adhesive tape were used to fix a sample. Water was evaporated from the SLM dispersion at room conditions. Before microscopic observations, the sample was coated with a thin layer of gold. Acceleration voltage of 5 kV was applied to record images at a magnification of 10,000×.

### 2.6. Entrapment Efficiency and Drug Loading

Percentage entrapment efficiency (EE%) and drug loading percentage (DL%) were determined using the total amount of drug added to the formulation and the amount of drug in microparticles according to the following formula:

(1) $$\mathrm{EE}\;\left(\%\right)=\frac{W\;\mathrm{initial}\;\mathrm{drug}-W\;\mathrm{free}\;\mathrm{drug}}{W\;\mathrm{initial}\;\mathrm{drug}\;}\times 100\%$$

(2) $$\mathrm{DL}\;\left(\%\right)=\frac{W\;\mathrm{initial}\;\mathrm{drug}-W\;\mathrm{free}\;\mathrm{drug}}{W\;\mathrm{lipid}}\times 100\%$$

where W_initial drug_ is the amount (g) of drug used for the formulation and W_free drug_ is the amount (g) of free drug detected in the aqueous phase after ultrafiltration. W_lipid_ stands for the weight (g) of the lipid matrix.

### 2.7. Characterization of API Distribution in SLM Dispersions

The total content of API in the formulations was determined as follows: 5.0 mL of methanol was added to 50 mg of API-loaded SLM; lipids were melted at 80 °C. After shaking and cooling to room temperature, methanol was refilled to 10.0 mL and the mixture was filtered. The concentration of API in the obtained solution was analyzed by HPLC.

The distribution of API between the aqueous phase, the interphase and the lipid core was analyzed as described below.

- **Aqueous phase (AP)**

The aqueous phase (AP) of the formulation was separated by using centrifuge ultrafiltration units—Microcon YM-100 (cut-off 100 kDa, Millipore, Bedford, MA, USA). The fraction of free drug dissolved in the aqueous phase of the SLM dispersion was determined by HPLC in a filtrate and expressed as a percentage of a total drug.

- **Interphase (IF) and methanolic phase (MP)**

To determine the amount of API localized at the interphase (IF), the procedure was as follows. An accurately measured amount of API-loaded SLM dispersion was suspended in methanol (1:5, *w*/*v*), shaken by vortex and centrifuged for 5 min at 3500 rpm. The sample of the supernatant was analyzed by HPLC in order to determine the fraction of the drug in methanolic phase (MP), where the sum of drug originating from the surface of the microspheres and from the aqueous phase (AP) was determined. The fraction of API localized in the interphase (on the lipid surface) was calculated as the difference between the amount of API found in the MP and AP—the amount determined in the aqueous phase was subtracted from the amount determined in the methanolic phase:

(IF) = (MP) − (AP)(3)

where (IF) is the amount of API located in the interphase, (MP) is the amount of substance determined in the methanolic phase and (AP) is the amount of substance determined in the aqueous phase.

- **Lipid core (LC)**

The amount of API incorporated in the lipid matrix (LC) was calculated as a difference between the total amount of API and the fraction found in the methanolic phase (MP).

### 2.8. In Vitro Drug Release Study

The in vitro drug release was investigated by mixing SLM directly with the acceptor fluid in the membrane-free system [13,18,19]. The appropriate quantity of SLM was suspended in 5.0 mL of sodium lauryl sulfate solution (0.5%) and incubated under agitation at 37 °C. Separate samples were prepared for each time point. At the predetermined time intervals, the aliquots of the SLM suspension were taken, filtered through 0.2 µm filter (cellulose acetate filters, Alchem, Torun, Poland) and diluted with methanol. The concentration of all APIs in the acceptor fluid was determined by HPLC method, as described in Section 2.9.

### 2.9. Analysis of Active Substances by HPLC

Concentration of all drug substances in tested samples was analyzed by reverse-phase high-performance liquid chromatography (RP-HPLC), using HPLC apparatus (Merck Hitachi, Tokyo, Japan) for KL analysis and Prominence LC-2030C 3D (Shimadzu Corporation, Kioto, Japan) for CsA, DNa and HC. The basic conditions for performing HPLC analysis for individual substances are presented in Table 2.

### 2.10. Statistical Analysis

All experiments were performed in replicates (*n* = 2–5). Presented results were reported as means and standard deviation (mean ± SD). Statistical analysis was performed using one-way analysis of variance (ANOVA), followed by post hoc Tukey HSD multiple comparison test. Statistical significance was set at *p* < 0.05. The results were processed by the statistical software Statistica (StatSoft program, Version 12, TIBCO Software, Palo Alto, CA, USA).

## 3. Results

### 3.1. Solubility of Drug Substances in Lipids

The melting point of the investigated lipids was as follows: Precirol 52–55 °C, Compritol 69–74 °C and stearic acid 70 °C [18,28,29]. Drug solubility was assessed in bulk-melted and also in solidified lipids. The physicochemical properties (molecular weight, log P) of the tested drug substances and the determined solubility in selected lipids are presented in Table 3. The solubility was determined semi-quantitatively. Presented in Table 3 concentrations of the drug substances dissolved in lipids are close to the saturated conditions, as the next portion of API introduced during the test did not dissolve completely. Despite this, the substances did not recrystallize after solidification in a thin lipid layer for a period of 7 days, as demonstrated by microscopic observations.

CsA, as a substance known for its good solubility in oils [30], was freely soluble in all lipids (10–33%). The effect of using the mixture of Precirol with Miglyol was negligible on the solubility of CsA, while a significant increase in solubility was observed in stearic acid (33%). The remaining tested substances showed a much lower solubility, increasing with an increase in log P of the drug substance (Table 3).

### 3.2. Characterization of SLM

The physicochemical properties of the SLM dispersions prepared by the same procedure have already been characterized in detail in previous publications [13,18,19,27].

All the tested SLM dispersions, irrespective of the lipid phase content (from 10% to 30% *w*/*w),* had a smooth liquid consistency.

**Figure 1:**
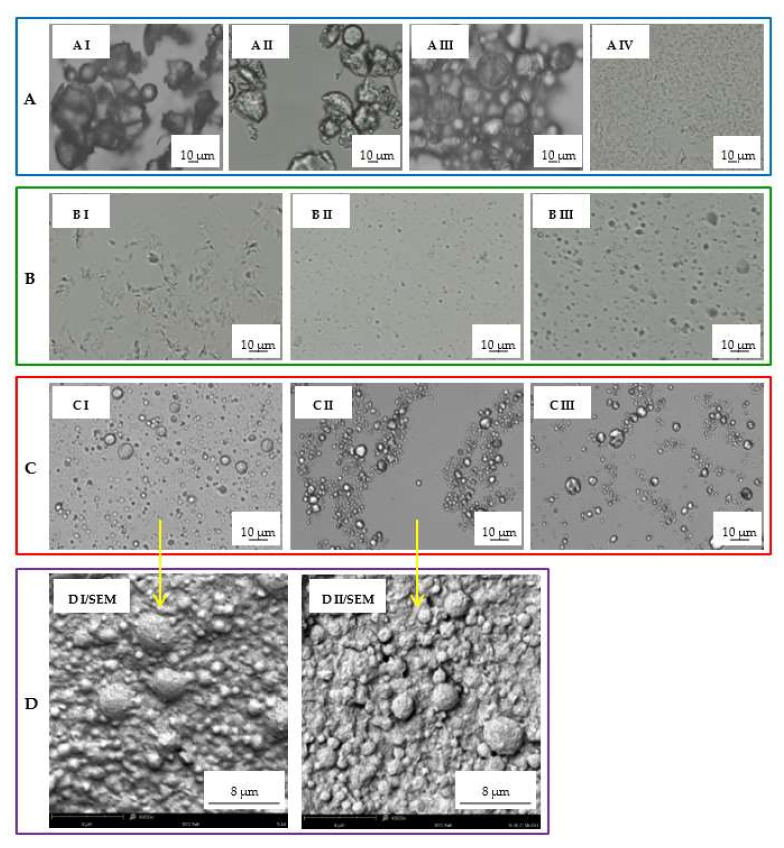
Optical microscope images of: (**A**) lipid (Compritol) bulk powder (AI), after 5 min of shaking with methanol at room temperature (AII), after 1 min of shaking with methanol at 60 °C (AIII) and in CH_2_Cl_2_ at room temperature (AIV); (**B**) 10% Compritol SLM dispersion after shaking (15 s) with 50% (BI), 16% (BII) and 4% (BIII) of methylene chloride; (**C**) 10% Compritol SLM dispersion (CI), after shaking (5 min on vortex) with methanol and with followed dispersion in water (CII) or in 3% polysorbate solution (CIII). (**D**) SEM images of 10% Compritol SLM dispersion before (DI/SEM) and after shaking (5 min on vortex) with methanol (DII/SEM).

In laser diffractometer measurements, the majority of the particles in SLM were in the range between 1 and 15 µm (Figure 1D), with small fraction (8%) outside of this range. This was confirmed with microscopic observations. Some increase (*d_0.9_* = 7.6 µm vs. *d_0.9_* = 11.1 µm) in the size of the microparticles was observed with increasing concentration of the lipid phase (10% vs. 30% *w*/*w*). The presence of APIs in the lowest from the tested concentrations did not affect SLM size, while the addition of API at a higher concentration (0.5–2.0%) resulted in an increase in size of the lipospheres from *d_0.9_* = 6.1 µm to *d_0.9_* = 7.0 µm.

The tested concentrations of DNa, KL and HC in SLM formulations were limited by API solubility. At higher concentrations than selected, crystallization of API was noticed (microscopic observations). Meanwhile, the concentration of CsA was selected on the basis of the previous in vivo studies in rabbits (SLM for ocular application [13]) and is not yet the highest concentration possible for incorporation in SLM.

The microscopic image of SLM particles showed that they were round-shaped and none of visible particle aggregates were noticed (Figure 1 CI). The particles with a regular, fairly smooth surface were observed in all formulations with the exception of lipospheres with stearic acid, which had a clearly uneven and irregular surface.

The pH of SLM dispersions after preparation was slightly acidic (5.5–6.5) or neutral (7.0–7.5) if a pH correction was performed. All formulations were characterized by a negative zeta potential, which most often amounted to approximately −30 mV (from −24 to −39 mV).

There was no effect of sterilization on the viscosity of liquid SLM formulations, assessed organoleptically. After sterilization, some reduction in the particle size was observed (from mean *d_0.9_* = 12.4 µm ± 2.9 to mean *d_0.9_* = 10.3 µm ± 3.1) or this parameter remained unchanged, regardless of the type and concentration of the lipid. At the same time, the change in the value of the zeta potential after sterilization, if any, was not more than 3 mV.

### 3.3. Assessment of the Solubility of the Lipid Matrix

**Figure 2:**
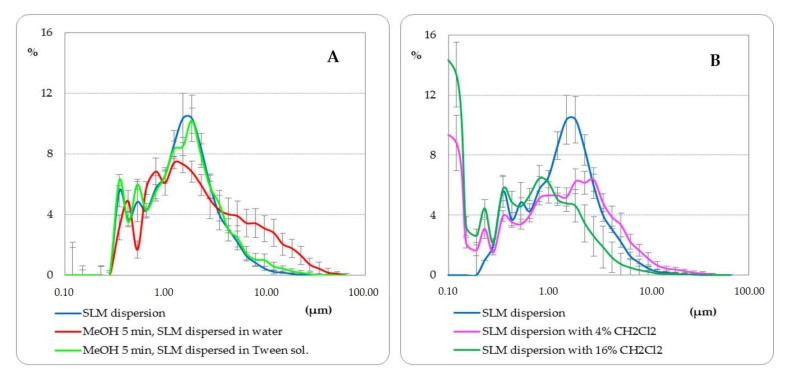
Microscopic particle size distribution (mean ± SD) in SLM dispersion (10% Compritol), untreated or treated with methanol (**A**) or methylene chloride (**B**).

Prior to the distribution studies, an initial multistage evaluation of the matrix lipid dissolution in various solvents was performed. Figure 1 and Figure 2 show the obtained results in the form of microscopic images and particle size distributions on the example of Compritol, because it was the basic lipid most often constituting the SLM matrix in the conducted research.

In the first step, the solubility of bulk lipids in methanol at room temperature and at 60 °C was assessed. As shown in Figure 1A, Compritol did not dissolve after 5 min of mixing with methanol at room temperature, as the shape of the particles did not change (AII vs. AI). Only at 60 °C (still below the melting point of the lipid) was a partial dissolution of the lipid particles observed (Figure 1AIII), gradually progressing with the mixing time. Glycerol palmitostearate (Precirol) behaved in the same way, while stearic acid dissolved slightly faster in methanol. The use of methylene chloride at room temperature resulted in fast and complete dissolution of the lipids (Figure 1AIV).

Furthermore, the effect of shaking SLM with methanol (final concentration of methanol about 80%) was assessed (see Section 2.5). In the microscopic images, no change in the size of the microspheres was observed, but SLM formed agglomerates and clusters of particles—Figure 1C (CII vs. CI). As a result, the particle size distribution measurements indicated an increase in the size of the lipospheres (Figure 2A). The use of the polysorbate solution to disperse SLM after shaking with methanol allowed the uniform dispersion of the microspheres in the microscopic image to be restored (Figure 1 CIII) and confirmed that the particle size distribution remained unchanged (Figure 2A). The absence of any changes in the structure and size of the microspheres as a result of shaking the SLM dispersion with methanol for 5 min is also confirmed by SEM images (Figure 1D). Differences in the structure and size of SLM, before and after shaking with methanol, in the SEM images were not observed in both placebo-SLM and drug-loaded SLM. As previously reported [18], it was not possible to identify clear differences in the surface appearance of microparticles with and without API using the SEM technique. In the presented studies, however, the lack of signs of dissolution of the lipid matrix was considered crucial, which is important from the point of view of the proposed method of API distribution determination.

The use of methylene chloride in a 1:1 ratio resulted in complete dissolution of the lipid matrix in the SLM dispersions (Figure 1 BI), similar to what was noted in bulk lipid (Figure 1 AIV). When methylene chloride was used in lower concentration (4%, 16%), the process of dissolution and degradation of the microspheres was slower and limited (Figure 1 BII and BIII). This effect was also clearly visible in the particle size distribution graphs (Figure 2B).

### 3.4. Entrapment Efficiency and Drug Loading

The determined content of the drug substance in the tested SLM dispersions was within the declared range of 100 ± 5%. The determined EE% and DL% values (Table 4) indicate the very wide range of these parameters (EE% from 24% to 100% and DL% from 0.5% to 19%) depending on the formulation. However, it should be also noted that for each drug substance, the total content in SLM was different, selected on the basis of its solubility in the lipids (Table 3). This approach also meant that crystallization/precipitation of API in the aqueous phase of SLM dispersions could be avoided.

In all formulations with Compritol, the CsA and KL entrapment efficiencies are close to 100%, regardless of API or lipid concentration. Changing the matrix lipid to stearic acid in SLM with CsA also did not influence the EE value, while the use of a mixture of solid and liquid lipid (PM) resulted in a reduction in EE to 71%. The use of a higher concentration of Tween 80 (5% instead of 3%) in SLM with 1% CsA increased EE by a few percent (from 94% to 100%), while in SLM with 0.1% KL, it had no effect. Significantly lower EE values (24–66%) were observed in SLM dispersions with DNa and HC, in which simultaneously a large fraction of API in the aqueous phase (76–34%) was determined. In formulations F7, F10, F12, F15, with the same composition (0.1% API, 10% Compritol, 3% polysorbate), EE decreased in the order: CsA > KL > DNa > HC (Table 4). The increase in API concentration resulted in slight changes in EE in the case of CsA and in a significant decrease in EE (from 67% to 24%) in SLM with DNa.

The obtained results indicate that the highest drug loading was achieved in the CsA formulations. The value of DL up to 20% (F2) was twice as high as predicted based on the solubility studies. At the same time, the high EE in formulation F2 (97%) suggests that it may not yet be the maximum achievable CsA concentration in the Compritol microspheres. In the case of other tested APIs, DL was very low (0.5–1%) which reflected the low total content.

No effect of thermal sterilization of SLM dispersion on DL was observed. The EE values also remained unchanged or the level of encapsulation increased by a few percent (only in formulation with HC was a slight decrease observed).

### 3.5. Characterization of API Distribution in SLM Dispersions

**Figure 3:**
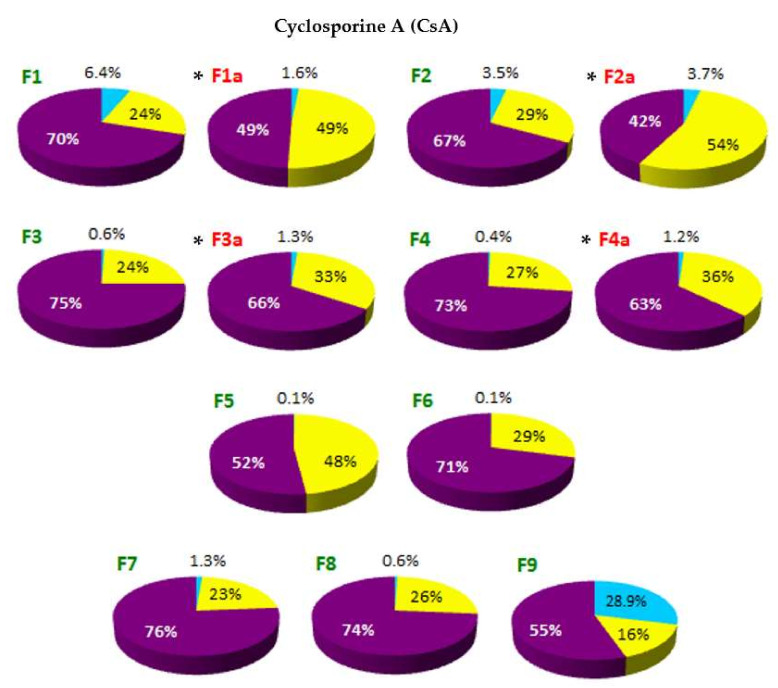
Distribution of cyclosporine (CsA) between the phases of SLM dispersions: aqueous phase (AP—blue color), interphase (IF—purple color) and lipid matrix (LC—yellow color). SLM dispersions were tested both before (formulations F1–F9) and after thermal sterilization (formulations F1a–F4a). (*)—formulations before and after sterilization, between which statistically significant differences were found (*n* = 3–5).

**Figure 4:**
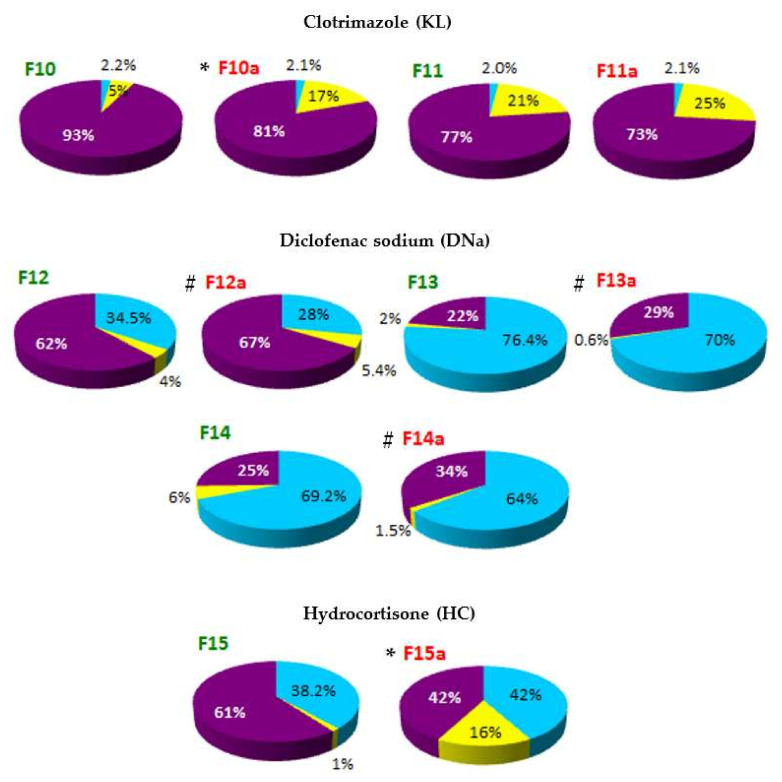
Distribution of clotrimazole (KL), diclofenac sodium (DNa) and hydrocortisone (HC) between the phases of SLM dispersions: aqueous phase (AP—blue color), interphase (IF—purple color) and lipid matrix (LC—yellow color). SLM dispersions were tested both before (formulations F10–F15) and after thermal sterilization (formulations F10a–F15a). (*)—formulations before and after sterilization, between which statistically significant differences were found (*n* = 3–5). (**#**)—formulations F12–F14 with DNa that were not statistically evaluated.

The localization of API in the phases of SLM dispersion—such as aqueous phase (AP), interphase (IF) and lipid matrix (LC)—determined on the basis of distribution studies, is shown in Figure 3 and Figure 4. Any precipitation of API in the aqueous phase was excluded by microscopic evaluation of the dispersions.

In the first stage, the amount of API in the aqueous phase, i.e., the fraction of the substance unbound with the lipid microspheres, was determined by ultrafiltration. An additional experiment was performed to check whether the remaining (beyond that determined in the aqueous phase) amount of API was incorporated into the SLM lipid cores, or if it was only bound to the lipid in the surface layers of the microspheres (interphase). For this purpose, the SLM dispersions were diluted with methanol and vortexed as described under Section 2.7. When SLM formulations were diluted with methanol, lipid matrix remained intact; therefore, the amount of API determined in the supernatant (marked as methanolic phase, MP) was the sum of the amount located in the aqueous phase and at the surface of the particles. Based on the above analysis, the amounts of API incorporated in the lipid matrix and located in the interphase (according to Equation (3)) were calculated.

On the basis of the obtained results, it was stated that in all tested formulations, except F13 and F14, the largest fraction of API (from 52% to even 93% of the total content) was located in the interphase—a surface layer of SLM, rich in surfactants. The remaining API is mostly found in the lipid matrix: the amount of API incorporated in the core of the SLM ranges from 21% to 48%. The exceptions are SLM with DNa (F12-F14), HC (F15) and one formulation with CsA (F9), where the lipid matrix is a mixture of Precirol and Miglyol. In these formulations, a significant amount of API (from 29% to even 76%) was identified in the aqueous phase as a fraction not bound in any way to the lipid matrix of SLM. In other formulations, the amount of API localized in the aqueous phase does not exceed 6.5%. Taking into account the amount of API that was incorporated in the lipid matrix, one can conclude that CsA and KL were incorporated much more easily in the lipid microparticles than DNa and HC.

Among the CsA formulations, systems with the same composition but different CsA concentration (1% and 2% in F1 and F2, respectively) showed a very similar API distribution: about 70% was found in the interphase and 24–29% in the lipid. This ratio also did not change when the lipid matrix was formed by stearic acid (F8) instead of Compritol. In F5 formulation with a very low concentration of CsA (0.1%), the API portion incorporated in the lipid core was almost 50%. A significant change was observed when the lipid matrix was formed by a mixture of solid and liquid lipids (F9). This significantly hampered the incorporation of CsA in the microspheres (both in the matrix and interphase), while increasing the API fraction in the AP to almost 30%. The increase in the concentration of the lipid phase from 10% to 30% in the dispersion, while maintaining the same CsA concentration (2%), also did not change the CsA ratio determined in individual phases. The lack of significant changes in the distribution of CsA in SLM following changes in the composition proves the stable affinity of this API for lipid of SLM. It also suggests that the maximum level of CsA inclusions has most likely not yet been reached.

In the case of the DNa and HC, their affinity to the lipid matrix can be considered significantly lower but very similar (F15 vs. F12). An increase in the DNa concentration in SLM from 0.1% (F12) to 0.5%, with the same concentration of the lipid phase (F13) or three times higher (F14), resulted in a doubling of the API fraction in the aqueous phase, while the amount determined in the interphase decreased from 62% to 22–25%. At the same time, the incorporation of DNa in the lipid matrix remained consistently low (2–6%).

Based on the results presented in Figure 3 and Figure 4, there is a clear evidence that the thermal sterilization process influences the distribution of API in the formulations (formulations before and after sterilization, between which statistically significant differences were found, are marked with (*) in Figure 3 and Figure 4). In the SLM with CsA, KL and HC, a clear reduction in the amount of API in the interphase was observed in favor of increasing the fraction incorporated in the lipid matrix. At the same time, neither significant changes in the amount of the substance determined in the aqueous phase nor precipitation were observed. The transfer of API from the interphase to the core of the lipospheres concerned a significant amount of the drug substance. In SLM with Compritol (10%), the CsA fraction incorporated in the lipid core increased even two-fold (F1–F2 vs. F1a–F2a, from 24–29% to 49–54%), but an even greater increase was observed in SLM with KL (F10, from 5% before sterilization to 17% after sterilization) and with HC (F15, 1% and 16%, respectively).

On the other hand, in the case of DNa as a result of sterilization, transfer of API from the aqueous phase to the interphase was noticeable—the amount of API, previously determined in the aqueous phase, decreased by about 5–7%, and no increase in DNa incorporated in the lipid matrix was observed.

### 3.6. In Vitro Drug Release Study

When comparing the results of the distribution study with the results of drug substance release from SLM, clear relationships can be found (Table 5).

At the beginning of the release process, a burst effect is observed, but the amount of API released during this time (1 h) generally does not exceed the amount of CsA determined in the interphase. The complete release of the amount of API determined in the methanol phase in the studied distributions takes at least 24 h or lasts longer (F1, F3, F6). The release of API incorporated in the lipid matrix is slow and, under the test conditions (without enzymes or factors causing the degradation of the lipid matrix), most often it is incomplete.

The changes in the distribution of CsA between the phases of the SLM dispersion due to thermal sterilization are also reflected in the results of the release test. Due to the increase in the API fraction incorporated in the lipid matrix, its release from the sterilized formulations is even slower, especially in the initial stage. Thus, the presented results of the release experiments confirm the determined distribution of the API in SLM.

## 4. Discussion

The main purpose of using SLM as a drug carrier is to ensure prolonged drug release, protection of the active substance or masking its taste. In each of the above cases, the effective incorporation of the drug molecules into the lipid matrix is essential to achieve the desired effect. The incorporation and thus the immobilization of drug molecules in the SLM lipid matrix is vital not only for the obtained release profiles, but above all for the stability of the formulation.

Even though the solid lipid particles (not only SLM, but mainly SLN formulations) are investigated for many years, the exact placement of drug molecules in the structures of the multi-compartment carrier is extremely difficult to accurately assess. There are many descriptions of the assumed, expected or predicted distribution of API in SLM or SLN [21,23,31,32], which, however, result from the adopted concepts of the multi-compartment, lipid dosage form, and not from the conducted analytical research.

The API distribution between individual phases of SLM dispersion depends on many factors such as the hydrophobicity of API, lipid type including its crystallization pattern, type and concentration of surfactant and method of SLM preparation [4,12,14]. In effect, it is not possible to predict the drug distribution and incorporation in lipid matrix only considering API properties and formulation composition. The rules that determine the drug distribution in different phases of dispersion have not been fully elucidated yet. In addition, too often non-standardized research methodology is used, which makes it difficult to compare the obtained results.

When the aqueous dispersion of lipid particles is considered as the drug carrier, as a rule the API is characterized as localized either in the lipid matrix or/and in the dispersing aqueous phase. This is presented with two parameters: entrapment efficacy (EE) and drug loading (DL) [3,4,6,7,15,16,33,34,35]. However, one should distinguish between the API incorporated in the lipid core (inclusion into the crystal lattice of the lipid matrix) and bound to the surface of lipospheres, in the so-called interphase, while being in contact with the lipid as well as the surfactant stabilizing the dispersion.

Based on the analysis of only these two parameters (EE and DL) in relation to the tested SLMs, the following conclusions could be presented (Table 4): (i) the tested drug substances can be classified into two categories—for CsA and KL the incorporation efficiency was very high (almost 100%), and DNa and HC were incorporated less effectively, but still the level of 24–66% was achieved, (ii) depending on the composition and concentrations significant incorporation efficiency was noted (DL values up to 20%). The similar high level of EE is very often presented in publications [3,4,5,16,24,33,36], but it should be clearly emphasized that such an assessment of SLM dispersions, based only on the EE and DL parameters, is misleading and incorrect. The surface-located API and the substance incorporated in the lipid core cannot be considered in the same way. As already mentioned, the surface-bound API performs in a different way than the fraction included in the lipid core, not contributing in the same extent in the required properties of SLM as a drug carrier. In dissolution tests, the API fraction located on the SLM surface usually results in a burst effect. Therefore, in scientific reports on SLM, it should be absolutely necessary to present a more detailed description of API distribution in the formulation. At this stage, choosing an appropriate method also becomes necessary to precisely determine the real degree of API incorporation in the lipid matrix.

Although a broad spectrum of analytical techniques was utilized to gather information about the properties of SLM formulations including qualitative imaging [12,15,21,22], the quantitative analysis of other API parameters apart from EE and DL is still uncommon.

Therefore, in this paper, a method of removing the API from the surface of lipid microspheres using methanol was proposed. Thus, the fraction generally considered as incorporated in SLM was separated into two fractions: bound in the interphase and incorporated in the lipid matrix core. The obtained results (Figure 3 and Figure 4) clearly indicate fundamental differences in the assessment based on the proposed distribution model and the parameters used so far (EE and DL, Table 4).

As a preliminary validation step, the potential of organic solvents to dissolve lipid particles was investigated using microscopic imaging (Figure 1). In addition, the effect of the solvents was additionally controlled by employing a microscope software and by measuring changes in the profiles of particle size distributions (Figure 2).

The obtained results were presented on the example of Compritol, because it was the lipid most frequently constituting the SLM matrix in the conducted research. In subsequent tests, it was shown that short-term (5 min) vortexing of the SLM dispersion with methanol (final concentration 80%) does not degrade the matrix, but allows the complete dissolution of API bound to the lipospheres surface. Additionally, the SEM evaluation of the tested SLMs before and after shaking with methanol (Figure 1D) confirms the lack of noticeable changes in the structure of the microspheres.

The proposed procedure included three parallel steps: a complete extraction of API with methanol after melting the lipospheres (total content analysis), ultrafiltration of undiluted SLM dispersion (fraction dissolved in the aqueous phase) and extraction of API from the interphase with methanol and separation of the methanol-aqueous phase by centrifugation (a sum of the content in the aqueous phase and in the interphase). The obtained results, presented in Figure 3 and Figure 4, lead to conclusions that are substantially different from those resulting from the interpretation of EE and DL values (Table 4).

Bearing in mind that the EE value does not only include the fraction of API from the aqueous phase, the results in Table 4 (especially for CsA and KL) suggest an excellent efficiency of incorporation of the tested drug substances into the microspheres. Meanwhile, the evaluation of the graphs in Figure 3 and Figure 4 clearly indicates that the aforementioned affinity is definitely weaker, and the majority of API in almost all tested formulations is located on the surface of the microspheres, instead of in the lipid matrix which could be suggested by high EE values. In fact, the fraction incorporated in the matrix core in most formulations does not exceed 30%, and in SLM with DNa and HC it is only 1–6%. Based on the results in Figure 3 and Figure 4, effective and permanent incorporation of API in the SLM lipid core seems to be much more difficult and limited. Although the CsA and KL fractions determined in the aqueous phase of the formulations was negligible, it was difficult to achieve high inclusion of these APIs in the lipid matrix, even if the results from the solubility study (Table 3) suggest this possibility. At the same time, the DNa and HC fractions corresponding to EE values (22–62%) are almost entirely surface-located. Despite the low concentrations of API (0.1–0.5%), it was not possible to successfully incorporate these APIs in the lipospheres.

Even with the appropriate affinity (lipophilicity) and solubility of API in the lipid, its incorporation into the lipid matrix may be limited and difficult. This is related to the effect of expulsion of the drug substance from the lipid matrix as a result of its crystallization (the substance does not enclose in the lipid matrix, but is removed outside of it). This phenomenon has already been described by other authors in many works [1,9,10,14,23,31,32,36,37]. As a result of the transformation of polymorphic lipids into SLM from unstable to more stable and ordered forms (conversion of higher energy α and β′ polymorphs to the stable β form), this effect may progress during storage and manifest as API precipitation in the aqueous phase. It is noteworthy, however, that the API removed from the matrix may localize in the interphase without the effect of precipitation outside the microspheres (in the aqueous phase). Such redistribution of API may result in obtaining a very stable SLM formulation with characteristic properties. As already demonstrated on the example of SLM dispersion with CsA, the formulations with a significant fraction of API localized in the interphase proved to be stable for at least 2 years [18]. The time-dependent distribution of API in selected SLM dispersions was also assessed. Long-term stability studies confirmed the lack of statistically significant differences in CsA distribution in SLM dispersions with CsA and Compritol (F1–F4), stored in the refrigerator for 2 years. The amount of CsA determined in AP, IF and LC remained unchanged and crystallization/precipitation of CsA in AP was not observed.

In SLM with the same composition of excipients (F7, F10, F12, F15), an increase in API solubility in lipid (Table 3) is consistent with an increase in API incorporation in the lipid matrix; however, this relationship is not proportional. Depending on the tested API, its concentration in lipospheres may also exceed the concentration determined in the solubility test (F2).

At the same time, the beneficial effect of the thermal sterilization process observed in some formulations deserves attention. This is important for drug formulations intended for parenteral or ocular administration, when sterility is a requirement. Even a fairly significant decrease in the interphase fraction and an increase in the amount of incorporated API may contribute to a greater stability of the dosage form or a longer release time. However, only the results of the proposed distribution studies allow the changes taking place in the matrix of lipospheres under the influence of the thermal sterilization process to be assessed, because the increase in the API fraction incorporated in the SLM core (LC fraction in F1a–F4a, F10a, F11a) is not provided by the EE or DL parameters, which do not change after sterilization (Table 4).

## 5. Conclusions

The presented studies show that the characterization of multi-compartment, lipid dosage form, such as SLM dispersions solely on the basis of EE and DL values, is insufficient and even misleading. This is because these parameters do not allow to distinguish the amount of API bound to the surface from the amount actually incorporated in the lipid cores. This approach indicates only the separation of the API between the aqueous phase and the lipid phase.

The proposed new distribution test method identifies not only the API fraction in the aqueous phase (AP), but also in the interphase (IF) and in the lipid matrix (LC). These characteristics of SLM provide a new tool helpful in the development of this dosage form and understanding its performance in vitro and in vivo.

This is especially important with respect to the results of the release study where the surface-located API is responsible for the observed burst effect. The obtained results also facilitate a critical assessment of the ease of incorporation of the drug into SLM and the manner of its encapsulation in the lipid matrix. The significant fraction of all tested APIs was located in the interphase, with much smaller fraction incorporated into the lipid matrix. That indicates—in contrast to the EE and DL results—limitations in the ease of API incorporation in SLM. Only the assessment of LC and IF parameters allows the influence of the sterilization process on the structure of SLM and the change in the API distribution within the lipospheres to be detected.

## Figures and Tables

**Table 1:** The composition (% *w*/*w*) of the investigated SLM formulations (F).

The Composition of SLM Dispersions										
F	DNa	HC	KL	CsA	Compritol	Stearic Acid	Precirol	Miglyol	Tween 80	Water Phase
F1	-	-	-	1.0	10.0	-	-	-	3.0	86.0
F2	-	-	-	2.0	10.0	-	-	-	3.0	85.0
F3	-	-	-	2.0	20.0	-	-	-	3.0	75.0
F4	-	-	-	2.0	30.0	-	-	-	3.0	65.0
F5	-	-	-	0.1	10.0	-	-	-	5.0	84.9
F6	-	-	-	1.0	10.0	-	-	-	5.0	84.0
F7	-	-	-	0.1	-	10.0	-	-	3.0	86.9
F8	-	-	-	1.0	-	10.0	-	-	3.0	86.0
F9	-	-	-	1.0	-	-	8.0	2.0	3.0	86.0
F10	-	-	0.1	-	10.0	-	-	-	3.0	86.9
F11	-	-	0.1	-	10.0	-	-	-	5.0	84.9
F12	0.1	-	-	-	10.0	-	-	-	3.0	86.9
F13	0.5	-	-	-	10.0	-	-	-	3.0	86.5
F14	0.5	-	-	-	30.0	-	-	-	3.0	66.5
F15	-	0.1	-	-	10.0	-	-	-	3.0	86.9

**Table 2:** HPLC analysis parameters for individual active substances.

HPLC Parameters	Active Substance			
	CsA	DNa	HC	KL
**Column**	LiChrospher 100 RP-18, 250-4	LiChrospher 100 RP-18, 250-4	LiChrospher 100 RP-18, 250-4	LiChrospher 100 RP-18, 250-4
**Mobile phase**	acetonitrile/water/ t-butyl methyl ether/ortophosphoric acid (520:430:50:1 *v*/*v*)	phosphate buffer/methanol (24:76 *v*/*v*)	acetonitrile/methanol/water (25:25:50 *v*/*v*)	phosphate buffer/methanol (20:80 *v*/*v*)
**Temperature**	80 °C	25 °C	25 °C	25 °C
**Flow rate**	2 mL/min	1 mL/min	1 mL/min	1.5 mL/min
**Wavelength**	210 nm	254 nm	254 nm	220 nm

**Table 3:** Solubility (*w*/*w*) of drug substances in tested lipids.

Active Substance			Lipid	Solubility of API in Lipid mg/g (% *w*/*w*)
MW (g/mol)	log P	Abbreviation		
318	4.5	DNa	Compritol	35 mg/g (3.5%)
362	1.6	HC	Compritol	<5 mg/g (<0.5%)
345	6.1	KL	Compritol	60 mg/g (6%)
1202	2.9	CsA	Compritol	100 mg/g (10%)
		CsA	Stearic acid	330 mg/g (33%)
		CsA	Precirol/Miglyol (4:1)	120 mg/g (12%)

**MW**: molecular weight.

**Table 4:** Entrapment efficiency (EE) and drug loading (DL) of tested SLM dispersions before and after thermal sterilization (means ± SD; *n* = 2–5).

Formulation	After Preparation		*After Thermal Sterilization*	
	EE	DL	*EE*	*DL*
F1	93.6 ± 6.3	9.4 ± 0.64	*98.4 ± 2.1*	*9.8 ± 0.21*
F2	96.5 ± 2.8	19.3 ± 0.55	*96.3 ± 3.5*	*19.3 ± 0.69*
F3	99.4 ± 1.2	9.9 ± 0.12	*98.7 ± 1.1*	*9.9 ± 0.11*
F4	99.6 ± 1.8	6.6 ± 0.12	*98.8 ± 1.5*	*6.6 ± 0.10*
F5	99.9 ± 3.1	1.0 ± 0.22	*–*	*–*
F6	99.9 ± 2.7	10.0 ± 0.26	*–*	*–*
F7	98.7 ± 3.7	1.0 ± 0.28	*–*	*–*
F8	99.4 ± 3.0	9.9 ± 0.31	*–*	*–*
F9	71.1 ± 8.1	7.1 ± 0.83	*–*	*–*
F10	97.8 ± 8.3	1.0 ± 0.55	*97.9 ± 6.5*	*1.0 ± 0.43*
F11	98.0 ± 3.7	1.0 ± 0.26	*97.9 ± 7.3*	*1.0 ± 0.51*
F12	65.5	0.7	*71.9*	*0.7*
F13	23.6	1.2	*29.8*	*1.5*
F14	30.8	0.5	*35.8*	*0.6*
F15	61.8 ± 5.7	0.6 ± 0.25	*58.2 ± 6.3*	*0.6 ± 0.28*

**EE**: mean % of total API ± SD; **DL**: mean % *w*/*w* of lipid matrix ± SD.

**Table 5:** Results of the drugs release study from the tested SLM dispersions with CsA before and after thermal sterilization in comparison to the API fraction localized in the interphase (mean ± SD; *n* = 3–5).

Formulation	IF (%)	API Release (%)	
		1 h	24 h
F1	70 ± 5.5	73 ± 4.5	79 ± 2.7
F1a	49 ± 3.9	41 ± 2.7	50 ± 7.8
F2	67 ± 6.4	71 ± 6.0	78 ± 4.3
F2a	42 ± 4.4	48 ± 2.8	59 ± 4.9
F3	75 ± 5.6	53 ± 3.3	71 ± 3.7
F3a	66 ± 3.2	47 ± 3.7	67 ± 5.3
F4	73 ± 5.6	45 ± 3.1	79 ± 5.5
F4a	63 ± 5.7	44 ± 4.9	74 ± 6.7
F5	52 ± 2.8	42 ± 2.8	62 ± 4.9
F6	71 ± 8.5	52 ± 3.3	67 ± 3.7

## Data Availability

Not applicable.
